# Comparison of 3 and 4 cycles of neoadjuvant gemcitabine and cisplatin for muscle-invasive bladder cancer: a systematic review and meta-analysis

**DOI:** 10.1186/s12885-023-11572-0

**Published:** 2023-11-06

**Authors:** Lanpeng Lu, Chaohu Chen, Hui Cheng, Hui Ding, Junqiang Tian, Hanzhang Wang, Zhiping Wang

**Affiliations:** 1 Institute of Urology, Lanzhou University Second Hospital, Lanzhou, China; 2 Key Laboratory of Gansu Province for Urological Diseases, Lanzhou, China; 3 Department of Pathology and Laboratory Medicine, UConn Health Farmington, Farmington, CT USA

**Keywords:** Bladder cancer, Neoadjuvant chemotherapy, Gemcitabine, Cisplatin

## Abstract

**Background:**

In muscle-invasive bladder cancer (MIBC), neoadjuvant chemotherapy (NAC) combined with radical cystectomy (RC) is critical in reducing disease recurrence, with GC (gemcitabine and cisplatin) being one of the most commonly used NACs. Different GC schedules have been used, but the best neoadjuvant regimen is still unknown. The clinical outcomes of 3 and 4 cycles of neoadjuvant GC are compared in this systematic review and meta-analysis to determine which is best for patients with MIBC.

**Methods:**

We searched PubMed, Embase, Web of Science, Cochrane Library, CBM, CNKI, WAN FANG DATA, and meeting abstracts to identify relevant studies up to March 2023. Studies that compared 3 and 4 cycles of neoadjuvant GC for MIBC were included. The primary outcomes were pCR, pDS, OS, and CSS. The secondary outcome was recurrence and SAEs.

**Results:**

A total of 3 studies, with 1091 patients, were included in the final analysis. Patients that received 4 cycles of GC had a higher pCR (OR = 0.66; 95% CI, 0.50–0.87; *p* = 0.003) and pDS (OR = 0.63; 95% CI, 0.48–0.84; p = 0.002) than those who received 3 cycles. Regarding recurrence rate (OR = 1.23; 95% CI, 0.91–1.65; p = 0.18), there were no appreciable differences between the 3 and 4 cycles of GC. Survival parameters such as OS (HR, 1.35; 95% CI, 0.86–2.12; *p* = 0.19) and CSS (HR, 1.06; 95% CI, 0.82–1.38; *p* = 0.20) were similar. Only one trial reported on the outcomes of SAEs. And there were no statistically significant differences in thrombocytopenia, infection rate, neutropenic fever, anemia, or decreased renal function between patients. The neutropenia of patients was statistically different (OR = 0.72; 95% CI, 0.52–0.99; *p* = 0.04).

**Conclusion:**

The 4-cycle GC regimen was superior to the 3-cycle regimen in only the pCR and pDS results. Survival and recurrence rates were similar between the two regimens. In both treatment regimes, the toxicity profile was manageable. However, due to the inherent drawbacks of retrospective research, this should be regarded with caution.

## Introduction

Bladder cancer is the 10th most common cancer worldwide, with 213,000 deaths and 573,000 new cases annually, and a quarter of these newly diagnosed bladder cancer cases had invaded the muscle tissue at the time of diagnosis [1].

Although radical cystectomy (RC) is the gold standard for the treatment of muscle-invasive bladder cancer (MIBC), half of the patients still experience distant metastasis after surgery [2]. Since the mid-1980s, multiple clinical studies have shown that bladder cancer has a good response to platinum-based combination chemotherapy. Randomized clinical trials (RCTs) and meta-analyses have shown that neoadjuvant chemotherapy (NAC) in MIBC can increase the overall survival rate (OS) of MIBC patients by 5–6% compared to direct RC [3–5]. Currently, patients with cT2-4aN0M0 (stage II or IIIA) are recommended to receive platinum-based combination neoadjuvant chemotherapy [6].

It was once thought that GC regimens (gemcitabine and cisplatin) and conventional MVAC regimens (methotrexate, vinblastine, doxorubicin, and cisplatin) have similar treatment response rates and survival rates, and GC is superior to MVAC in terms of safety and tolerability [7]. However, the VESPER trial recently came to a different conclusion: 6 cycles of dose-dense MVAC were more effective than 4 cycles of GC in terms of progression-free survival (PFS) and OS. However, dd-MVAC caused more severe asthenia and gastrointestinal side effects than GC [8, 9]. Because of their good therapeutic effects (Table 1), dd-MVAC and GC have become more widely used in clinical treatment. Both protocols were also written into the National Comprehensive Cancer Network guidelines in 2020 [10].

**Table 1:** Recent comparison studies of the dd-MVAC and GC regimens

Author(s) (Year)	Study Design	NAC Regimen	Sample Size	pCR,n (%)	pDS,n (%)	OS,HR (95% CI)	CSS,HR (95% CI)	Toxicity
van de Putte EE et al. 2016 [11]	Retrospective	GC 4 cycles	51	16(31.4)	22(43.1)	–	–	Grade 3–4 toxicity rates related to dd-MVAC (32%) and GC (44%) were similar (p = 0.202).
		dd-MVAC 4 cycles	80	23(28.8)	30(37.5)	–	–	
Zargar H et al. 2017 [12]	Retrospective	GC 3–4 cycle	219	32(14.6)	98(44.8)	2.07(1.25–3.42)	2.31(1.29–4.13)	–
		dd-MVAC 3–4 cycle	100	28(28.0)	69(69.0)	1	1	
Peyton CC et al. 2018 [13]	Retrospective	GC 3–4 cycle	204	50(24.5)	92(45.1)	1	–	–
		dd-MVAC 3–4 cycle	46	19(41.3)	24(52.2)	0.42(0.17–1.06)	–	
Ruplin AT et al. 2020 [14]	Retrospective	GC 3–4 cycle	76	19(25.0)	38(50.0)	–	–	–
		dd-MVAC 3–4 cycle	33	7(21.2)	13(39.4)	–	–	
Pfister C et al. 2021 [9]	Prospective	GC 4 cycle	198	71(35.9)	98(49.5)	–	–	The hematological toxicities rate in dd-MVAC (52%), and GC (55%), were comparable. Gastrointestinal (GI) grade ≥ 3 disorders were more frequently observed in the dd-MVAC arm (p = 0.003), as well as asthenia of grade ≥ 3 (p < 0.001).
		dd-MVAC 6 cycle	199	84(42.2)	126(63.3)	–	–	

–, not available

Currently, the GC regimen used in clinical practice has both a 21-day and a 28-day treatment cycle, as well as 3 or 4 cycles. The specific drug cycle, dose intensity, and total dose of different GC regimens are not the same. There are few studies that compare and analyze the clinical outcomes of different GC-neoadjuvant chemotherapy regimens. Therefore, the optimal GC neoadjuvant chemotherapy regimen has yet to be determined, and the optimal number of GC-neoadjuvant chemotherapy cycles remains uncertain. This systematic review and meta-analysis aims to compare the clinical results and toxicity among patients who received 3 or 4 cycles of neoadjuvant GC to guide clinical practice.

## Materials and methods

### Literature search and data extraction

This systematic review has been registered on PROSPERO (CRD42023409693). We followed the Preferred Reporting Items for Systematic Reviews and Meta-Analyses (PRISMA) statement and the recommendations of the Cochrane Collaboration (http://www.prisma-statement.org/) for reporting preferences during the conduct of this meta-analysis.

We searched PubMed, Cochrane Library, Web of Science, Embase, the Chinese Biomedical Database (CMB), the Chinese National Knowledge Infrastructure (CNKI), and WAN FANG DATA up to March 2023 for pertinent studies that contrasted 3 and 4 cycles of the neoadjuvant GC for MIBC. The terms “bladder cancer,“ “bladder carcinoma,“ “neoadjuvant chemotherapy,“ “gemcitabine,“ and “cisplatin,“ as well as pertinent variations of these terms, are pertinent Medical Subject Headings. ((gemcitabine) OR (cisplatin)) AND ((bladder cancer) OR (bladder carcinoma)) AND ((neoadjuvant chemotherapy)) were used to create the search algorithm.

The titles and overviews of the retrieved articles were independently reviewed by two reviewers based on their inclusion criteria. A third reviewer settled any disagreements within the two reviewers on the data extracted. To get any missing data, the authors were contacted. The missing data was not included if they didn’t respond to our persistent inquiries. We chose the most recent article that contained the most recent information when the very same study was given more than once.

As a meta-analysis, ethical approval from an institutional review board or ethics committee was not required for this study.

### Inclusion criteria and study eligibility

The eligibility of each study was determined based on the PICOS framework (par-ticipants, interventions, comparators, outcomes, and study design) to determine their suitability for inclusion [15]. Participants: patients with confirmed MIBC by biopsy who have undergone systemic neoadjuvant GC therapy; Interventions: MIBC patients who have received three cycles of systemic GC; Comparison group: MIBC patients with similar characteristics who have received four cycles of systemic GC; Outcomes: Comparison of oncological outcomes, including pathologic downstaging (pDS), pathologic complete response (pCR), overall survival (OS), cancer-specific survival (CSS), recurrence, and severe adverse events (SAE); Study design: There were no restrictions on study design, and randomized controlled trials and non-randomized observational studies were included in the analysis.

The primary outcomes were pCR, pDS, OS, and CSS. The secondary outcome were recurrence and SAEs. After surgery, a pathological investigation was used to determine both pCR and pDS. The TNM classification for pT0N0 or ypT0N0 in the final pathology was used to determine pCR. A lower pathologic stage than the preoperative clinical phase, or down staging to a non-muscle-invasive illness, was used to define pDS. The interval between the date of surgery and cancer-specific mortality or death from any cause, respectively, was used to determine CSS and OS. According to the National Cancer Institute’s (NCI) Common Terminology Criteria for Adverse Events, SAEs are those with a rating of ≥ 3.

### Quality assessment

The quality assessment was independently conducted by two reviewers using the Newcastle-Ottawa Scale [16]. The Newcastle-Ottawa scale has three main assessment categories: exposure, comparability, and selection. Studies can receive up to nine points. A final rating of six points or higher denotes high quality.

### Statistical analysis

The pCR, pDS, SAEs, and recurrence are represented as two dichotomous variables. In order to determine their 95% confidence intervals (CIs) and the odds ratios (ORs), the frequency of the events was subtracted. The OS and CSS outcomes were represented by hazard ratios (HRs) and their 95% confidence intervals (CIs). The results of studies with survival curve results were gathered from the Kaplan-Meier curve using published methods [17].

Between-study heterogeneity was assessed using chi-square and *I*2 tests. A Cochran Q statistic *p*-value < 0.05 or an *I*2 statistic > 50% was used to indicate statistically significant heterogeneity between trials [18].

According to the degree of heterogeneity, fixed-effect models or random-effects models were used to calculate summary statistics. For the initial analysis, a fixed-effects model was used, and a random-effects model was used for confirmatory analysis if significant heterogeneity was present [19]. When at least ten studies were involved in a particular outcome, a funnel plot was recommended for small-study effects assessment, according to version 6.3 of the Cochrane Handbook for Systematic Reviews of Interventions. However, only three studies met the inclusion criteria for this review. Review Manager 5.3 was used for meta-analysis.

## Results

### Literature search and characteristics

**Fig. 1:**
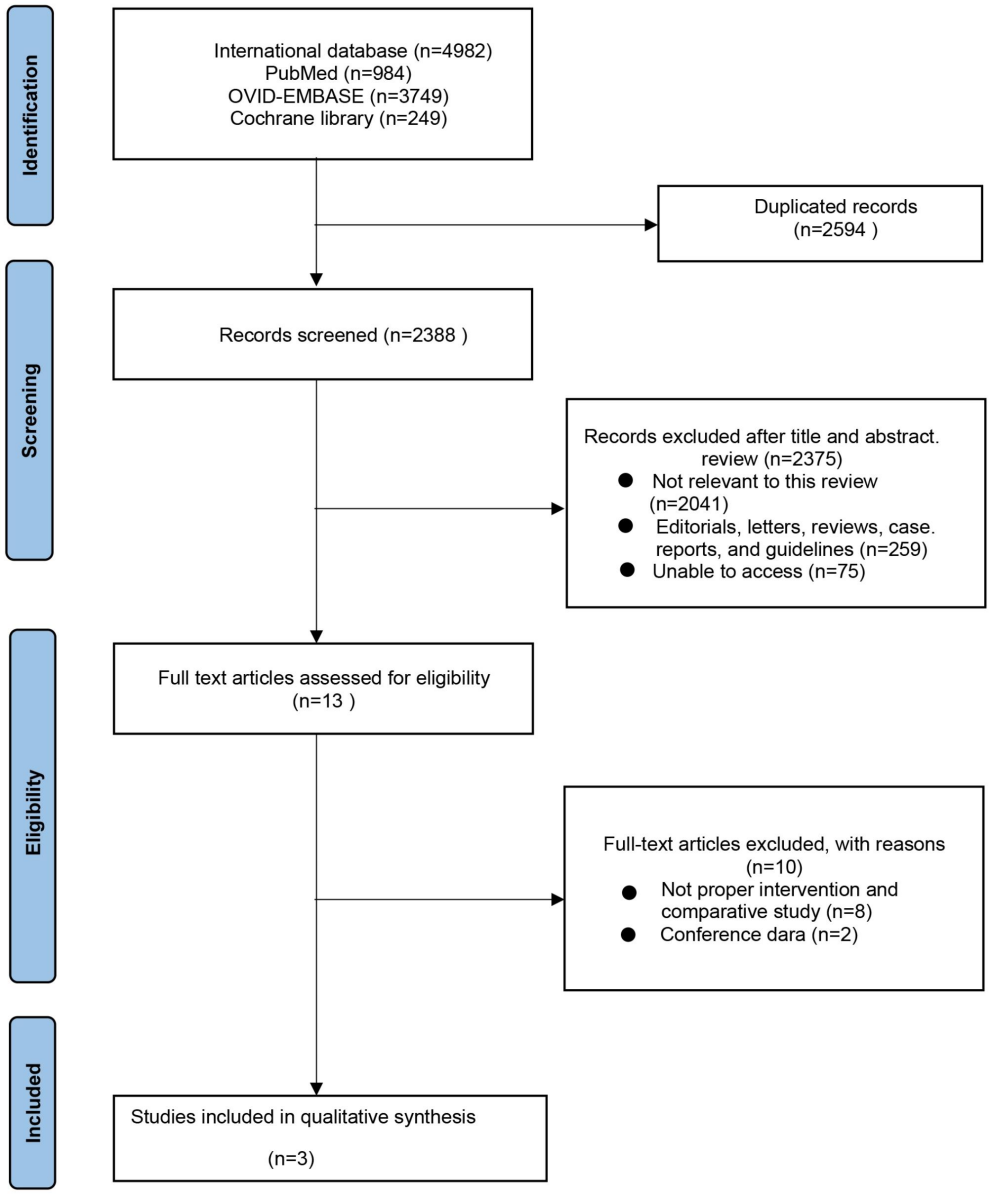
Systematic reviews and meta-analysis (PRISMA) flow chart illustrating the selection process of the studies

A flow chart of study selection according to PRISMA guidelines is presented in Fig. 1. The preliminary database search yielded 4982 studies (984 in PubMed, 3749 in OV-ID-EMBASE, and 249 in the Cochran library). After deleting duplicates, 2338 of these studies were left for review. Following a review of the titles and abstracts, 13 articles were disqualified. Using predetermined inclusion criteria, ten studies were examined. Two studies [20, 21] that used MVAC were disregarded. Additionally, two conference abstracts [22, 23] were further excluded. Three studies [24–26], with a total of 1091 patients, were included in the final analysis. Characteristics and interventions for them are presented in Table 2. All studies were retrospective case-control studies. Patients who had received neoadjuvant GC and had been diagnosed with MIBC were enrolled in all trials.

**Table 2:** Characteristics and interventions of studies included in the meta-analysis

Authors	Year	Country	Study Design	Sample size			Age (median, years)		Gender (male, %)		Outcomes	median follow-up-time
				3 cycles	4 cycles	Total	3 cycles	4 cycles	3 cycles	4 cycles		
Holmsten K et al. [24]	2022	Sweden and Denmark	Retrospective multi-institutional	251	455	706	67 (44–80)	65 (34–79)	184 (73)	324 (71)	pCR, pDS, OS, CSS, SAEs, recurrence	3 cycles: 43.2 months 4 cycles: 32.4 months
Aydin AM et al. [25]	2022	USA	Retrospective single-institutional	107	59	166	66.0 (60.0-72.5)	65.0 (58.5–71.0)	80 (74.8)	44 (74.6)	pCR, pDS, OS, CSS	18.6 months
Ferro M et al. [26]	2022	Italy	Retrospective multi-institutional	160	59	219	66 (59–72)	66 (60–74)	133(83.1)	48(81.4)	pCR, OS, CSS, recurrence	76 months

OS, overall survival; SAEs, severity adverse events; pCR, pathologic complete response; pDS, pathologic down-staging rate; CSS, cancer-specific survival

### Quality assessment

The Newcastle-Ottawa scale was used to assess the quality of the nonrandomized studies, as shown in Table 3. Three studies received a 7-point rating, indicating that they were of high quality.

**Table 3:** Results of quality assessment of nonrandomized studies using the Newcastle–Ottawa scale

Author(s) (Year)	Selection (4)				Comparability (2)	Exposure (3)			Total score
	Adequate definition of cases	Representativeness of cases	Selection of controls	Definition of controls	Control for important factor or additional factor	Ascertainment of exposure	Same method of ascertainment for cases and rate controls	Non-Response rate	
Holmsten K et al. 2022	1	1	0	1	2	1	1	0	7
Aydin AM et al. 2022	1	1	0	1	2	1	1	0	7
Ferro M et al. 2022	1	1	0	1	2	1	1	0	7

### Pathologic complete response rate

**Fig. 2:**
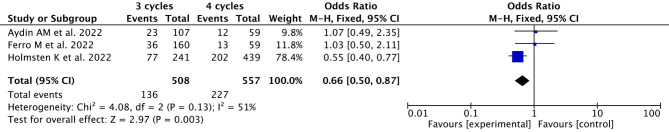
Forest plots of pathologic complete response rates

For pCR, 3 studies with 1,065 patients were eligible for this analysis. The pCR was observed in 26.8% (136/508) of the 3 cycles of GC and in 40.1% (227/557) of the 4 cycles of GC (Table 1). An analysis was conducted, as shown in Fig. 2, and the pCR rate was higher in the 4 cycles of the GC group (OR = 0.66; 95% CI, 0.50–0.87; *p* = 0.003), and heterogeneity was not found across studies (*I*2 statistic, 51%; Cochran Q statistic, *p* = 0.13).

### Pathologic down staging rate

**Fig. 3:**
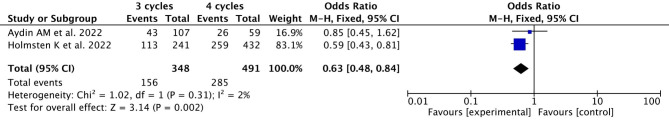
Forest plots of pathologic down staging rates

For pDS, 2 studies of 839 patients were eligible for analysis. In the 3 and 4 cycle regimens, the pDS rates were 44.8% (156/348) and 58.0% (285/491), respectively (Table 2). According to Fig. 3, the analysis was carried out. The two regimens had significantly different pDS rates (OR = 0.63; 95% CI, 0.48–0.84; *p* = 0.002), and there was no study heterogeneity (*I*2 statistic, 2%; Cochran Q statistic, *p* = 0.31) between them.

### Overall survival

**Fig. 4:**
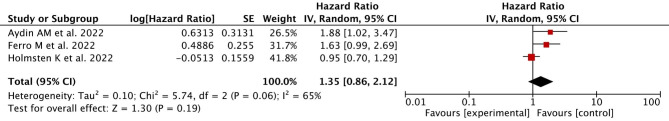
Forest plots of overall survival

OS results between the two treatment regimens are shown in Fig. 4. The OS analysis included three trials, and the findings showed that there was no statistically significant difference in OS across the arms (HR, 1.35; 95% CI, 0.86–2.12; *p* = 0.19). The studies were statistically different from one another (*I*2 statistic, 65%; Cochran Q statistic, *p* = 0.06).

### Cancer-specific survival

**Fig. 5:**
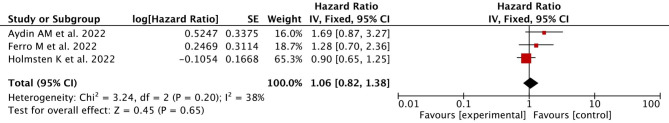
Forest plots of cancer-specific survival

Three studies reported CSS outcomes shown in Fig. 5. There was no significant difference between 3 cycles and 4 cycles of GC (HR, 1.06; 95% CI, 0.82–1.38; *p* = 0.20). Heterogeneity was not present (*I*2 statistic, 38%; Cochran Q statistic, *p* = 0.20).

### Recurrence

**Fig. 6:**
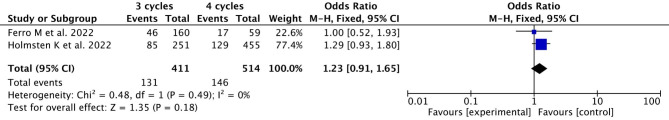
Forest plots of recurrence

The recurrence analysis shown in Fig. 6 could be performed on two studies with 925 patients. In 3 cycles, 31.8% of recurrences (131/411) and in 4 cycles, 28.4% of recurrences (146/514) occurred. The rate of recurrence did not differ substantially between the two treatment plans (OR = 1.23; 95% CI, 0.91–1.65; *p* = 0.18), and there was no heterogeneity among results from the trials (*I*2 statistic, 0%; Cochran Q statistic, *p* = 0.49).

### Sever adverse events

**Fig. 7:**
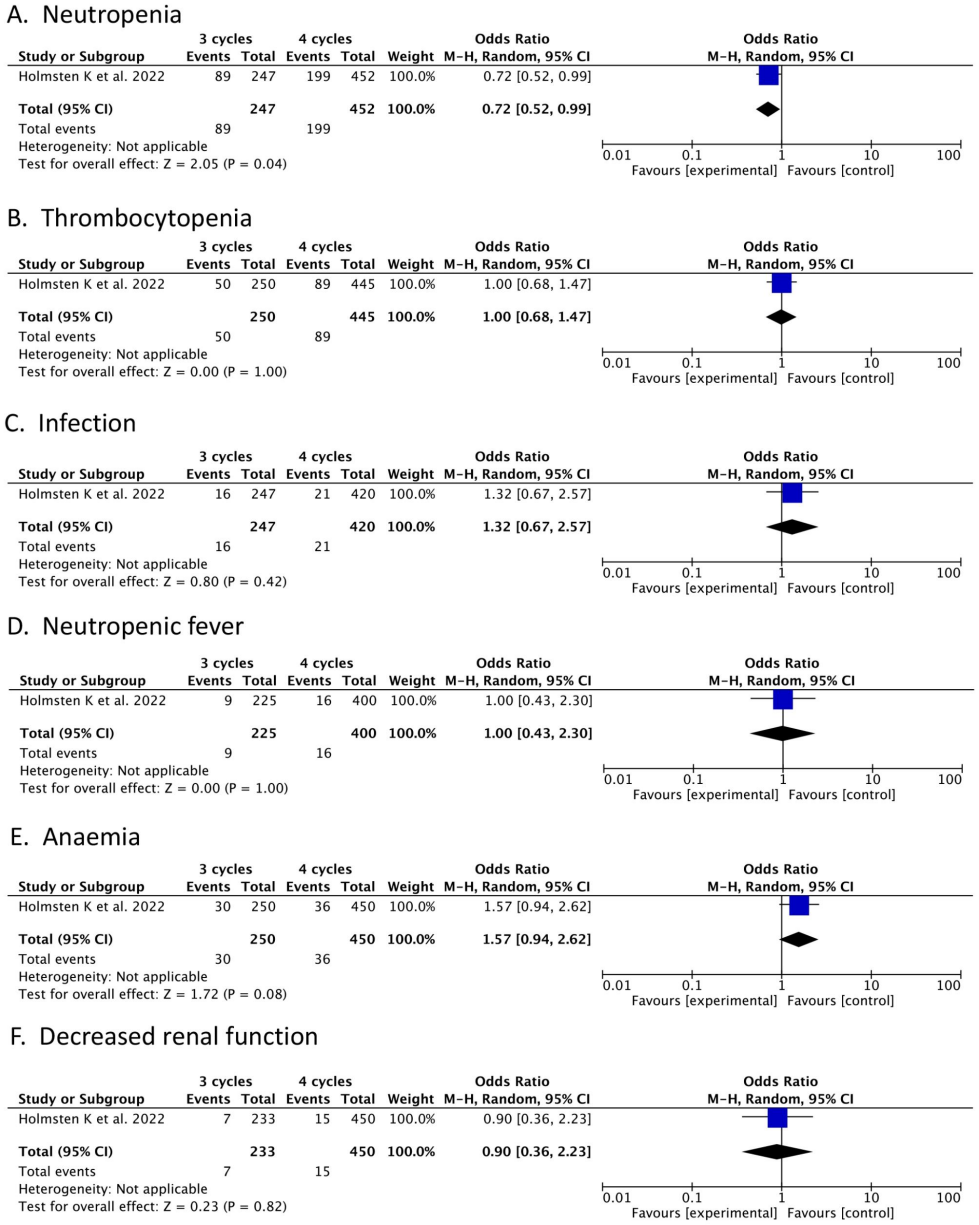
Forest plots of Sever Adverse Events. (**A**) Neutropenia. (**B**) Thrombocytopenia. (**C**) Infection. (**D**) Neutropenic fever. (**E**) Anemia. (**F**) Decreased renal function

Only one trial reported on the outcomes of SAEs. We conducted six analyses of SAEs, including neutropenia, thrombocytopenia, infection, neutropenic fever, anaemia, and decreased renal function, as shown in Fig. 7. The neutropenia of patients was statistically different (OR = 0.72; 95% CI, 0.52–0.99; *p* = 0.04) (Fig. 7(A)). There were no statistically significant differences in thrombocytopenia between patients (OR = 1.00; 95% CI, 0.68–1.47; *p* = 1.00) (Fig. 7(B)). The infection rate among patients did not differ statistically. (OR = 1.32; 95% CI, 0.67–2.57; *p* = 0.42; Fig. 7(C)). The rate of neutropenic fever among patients was not statistically different (OR = 1.00; 95% CI, 0.43–2.30; *p* = 1.00) (Fig. 7(D)). There was no difference in anemia (OR = 1.57; 95% CI, 0.94–2.62; *p* = 0.08) (Fig. 7(E)). Decreased renal function showed no differences (OR = 0.90; 95% CI, 0.36–2.23; *p* = 0.82) (Fig. 7(F)).

## Discussion

Since the Southwest Oncology Group reported positive results with NAC for MIBC using MVAC in 2003 [27], the exploration of NAC regimens has gradually diversified. With the advancement of Phase II and III clinical trials for MIBC NAC, the GC regimen and the MVAC regimen have gradually emerged from many regimens because of their advantages of both less toxic side effects and good tumor treatment outcomes, becoming the most commonly used NAC regimen for MIBC [10, 28]. For radically resectable MIBC, neoadjuvant chemotherapy combined with radical cystectomy and pelvic lymphadenectomy is currently the gold standard of treatment. In NAC for MIBC, there was no significant difference in response rate or survival between GC and MVAC, the two most common preoperative chemotherapy regimens. According to a large, multicenter trial report of 935 patients, the incidence of pT0N0 disease was 23.9% in patients receiving GC regimen chemotherapy compared with 24.5% in patients receiving MVAC regimen chemotherapy (p = 0.2) [29]. GC, however, has a higher safety and tolerability profile compared with MVAC. The VESPER trial study showed that dose-dense MVAC caused more severe fatigue and gastrointestinal side effects than GC in perioperative chemotherapy. Despite its limitations, the study showed that 6 cycles of dose-dense MVAC were more effective in terms of PFS and OS than 4 cycles of GC [9]. Therefore, GC regimens are among the most widely used regimens in clinical practice.

In some studies, optimal NAC regimens were defined as 3 cycles of cisplatin-based regimens, whereas suboptimal NAC was defined as 1–2 cycles of cisplatin-based or non-cisplatin-based regimens. According to a retrospective multicenter study, optimal NAC had better RFS and OS compared with suboptimal or no NAC after propensity score matching [30]. Another study showed a higher rate of complete pathological response in the optimal NAC group (30.8%) than in the suboptimal group (15.6%) (p = 0.03) [31]. Similar conclusions were reached by Zhegalik AG et al [32]. One of the highest downstaging (pT2N0) rates to date (57%) has been reported in MIBC patients in a study of 6 cycles of a neoadjuvant GC regimen; however, 39% of these patients had to down- titrate because of toxic side effects. Only 67% of patients completed the planned six cycles [33]. Thus, NAC has significant differences between clinical trial design and actual clinical practice. Although long courses of NAC therapy have a more pronounced downstaging effect, the delay of surgery and chemotherapy-related toxicity may still negatively impact survival, so the optimal cycle of preoperative GC should be considered comprehensively.

In the randomized Phase 3 studies comparing GC and MVAC regimens, the GC regimen was set as a 28-day regimen, and cisplatin dosing in the MVAC regimen was consistent with the GC regimen, which reduced between-group differences and improved comparability between the GC and MVAC regimens. The GC regimen showed significantly fewer side effects than the MVAC regimen, and since then, GC has replaced MVAC as the standard NAC regimen for MIBC. In addition, 3-cycle and 4-cycle GC regimens have also been used in the clinical treatment of advanced urothelial carcinoma [7]. Currently, the NCCN guidelines recommend a 4-cycle GC chemotherapy regimen as the NAC regimen for MIBC [34], while the AUA and EAU guidelines lack specific recommendations regarding the optimal number of cycles of GC chemotherapy due to limited evidence [6, 35]. Because of this, 3–4 cycles of the GC regimen continue to be used in clinical practice, but few studies have reported differences in clinical treatment outcomes between 3 and 4 cycles of chemotherapy [36]. Although GC is one of the most commonly used neoadjuvant regimens, knowledge of its optimal number of neoadjuvant GC cycles remains limited in the academic community.

Recently, two multicenter retrospective studies have reported differences in clinical treatment outcomes between three cycles versus four cycles of neoadjuvant chemotherapy in MIBC patients, but the results are conflicting. Patel et al. [21] reported the clinical outcome of patients receiving cisplatin-based neoadjuvant chemotherapy (GC, MVAC, gemcitabine, carboplatin, and other non-cisplatin-based combinations). Some patients changed their original treatment plan because they could not tolerate the toxic side effects caused by chemotherapy, and about 30% of patients who originally planned to receive 4 cycles of NAC completed only 3 cycles of chemotherapy. Patients who completed 3 cycles of cisplatin-based neoadjuvant chemotherapy showed similar pathological responses and short-term survival compared with those who completed 4 cycles of chemotherapy. However, the study by D’Andrea et al. [20], showed that patients receiving 4 cycles of NAC (GC, MVAC, and dose-dense MVAC) had a higher pCR rate (28% vs. 21%, p = 0.02) and OS (HR 0.68; 95% CI 0.49, 0.94; p = 0.02) than patients receiving 3 cycles of NAC (GC, MVAC, and dose-dense MVAC).

In retrospective analyses, the pCR rate with NAC for MIBC ranged from 20–30% [36–38]. However, none of these reports have investigated and analyzed different NAC cycle numbers. A single-center retrospective study found that patients who received 4 cycles with dose reduction or 3 cycles without dose reduction had a higher rate of pathological response than patients who received fewer cycles of chemotherapy [39]. Unfortunately, this study did not directly compare the differences between 3 and 4 cycles of chemotherapy and did not correlate the number of cycles administered with survival for further studies and analyses. Our study showed that patients receiving the 4-cycle GC regimen had a higher rate of pathological response than those receiving the 3-cycle GC regimen. The proportion of pT0N0 was greater in patients receiving the 4-cycle GC regimen, which we speculate may be due to the higher cisplatin dose intensity and cumulative dose in the 4-song cycle regimen. Similarly, a recently published meta-analysis indicated that the dd-MVAC regimen had a more pronounced pathological downstaging effect because its total cisplatin dose and dose intensity were higher than those of the GC regimen [40].

Normally, the gold standard for evaluating the effectiveness of a therapy is often a pathological examination, and pCR is associated with a long-term survival benefit. Patients who achieve a pCR, which is defined as ypT0 ypN0 or ypT0/is ypN0, have improved survival [41]. In present study, there was no further corresponding improvement in CSS or OS in the patient group treated with 4 cycles of neoadjuvant GC, despite the positive downstaging efficacy. We speculate that the following reasons may explain the discrepancies among the available studies: (1) The OS and CSS outcomes of bladder cancer NAC patients are influenced by the subsequent treatment, as the disparities in OS and CSS between 3 and 4 rounds of chemotherapy can be hidden by RC. The study found that more than 20% of cT0 patients who received bladder-sparing therapy suffered recurrence or died during follow-up [42]. GC can eliminate micrometastases, reduce tumor volume, and lower invasiveness, thereby increasing the success rate of surgical removal [43, 44]. However, both 3-cycle and 4-cycle chemotherapy patients received RC treatment after GC. The scope of surgical resection of RC in MIBC patients was unaffected by the cycles of GC, and the extent of surgical removal is closely associated with patients’ post-operative recovery and long-term survival [45]. The surgical scope for both 3-cycle and 4-cycle chemotherapy patients is the same, significantly reducing the possibility of the primary lesion being the source for tumor recurrence and metastasis and eliminating the differences in OS and CSS indicators between 3-cycle and 4-cycle chemotherapy patients. (2) 3-cycle GC may already be effective in eliminating existing micrometastases, and 4-cycle GC might not enhance this efficacy. Although the effect of NAC treatment on micrometastases is positive, its reduction effect is not the same for tumors with different stages [46]. The study found that because cT3 tumors are larger and more infiltrative, with a higher rate of nodal and distant dissemination, the relative ratio of micro metastatic disease is higher in cT3 tumors than in cT2, whereas the relative ratio of untreatable micro metastatic nodal dissemination peaks in cT4a tumors [47]. As a result, the effect of NAC on OS would be greatest in cT3 tumors [37]. Based on this, we can infer that when tumor staging is relatively low, the reduction effect of 3-cycle and 4-cycle GC treatments is similar, and the effect on CSS and OS outcomes is not much different for these patients.

The sensitivity of tumors to cisplatin is the most critical factor determining the therapeutic effect for cancer patients; cisplatin sensitivity is far more important than the final cumulative dose and dose intensity of cisplatin [48, 49]. Some patients are insensitive to cisplatin [50, 51], and even higher doses of GC combination chemotherapy cannot completely eliminate drug-resistant micrometastases. To eradicate these micrometastases, the combination of GC with other antitumor drugs can be considered to improve the efficacy of neoadjuvant chemotherapy. For example, this can be accomplished by combining immunotherapy with immune checkpoint inhibitors (ICIs) [52], targeted therapies such as inhibitors of fibroblast growth factor receptor (FGFR) [53], or antibody-drug conjugates (ADCs) [54]. Recently, it was suggested that a less stringent follow-up regimen could be applied to patients with pCR after RC [55]. However, pT0 did not mean a patient was free of bladder cancer [56]. Previous studies have shown that pT0 patients have lower disease-specific mortality; nonetheless, there are still pT0 patients who die from bladder cancer [57]. As a result, this should be considered during treatment planning, and patients should be closely monitored during their survivorship.

Four cycles of GC chemotherapy were associated with a higher incidence of grade 3/4 adverse events, particularly hematologic toxicity. According to our findings, there were no statistically significant differences in thrombocytopenia, infection rate, neutropenic fever, anemia, or decreased renal function between 3 and 4 cycles of GC. Four cycles of GC chemotherapy were found to be related to a higher incidence of neutropenia. This finding indicates the use of granulocyte colony stimulating factor (G-CSF) prophylaxis as part of the 4-cycle GC regimen should probably be considered routine. However, only one of the included studies mentioned the adverse events; inadequate data may have hampered this assessment. We reviewed relevant studies regarding adverse effects of neoadjuvant GC regimens; a total of 5 studies showed toxicity data, of which 3 were retrospective studies [11, 58, 59], 1 RCT [60], and 1 prospective study [61]. Anemia, leukopenia, thrombocytopenia, vomiting, and diarrhea are among the most frequently reported side effects in the literature (Table 4). Grade 3/4 anemia has been reported in 0–32% of patients. The frequency of vomiting in patients has been reported to be 2.6–40% in the literature. Differences in the frequency of adverse reactions were explained by the different patient characteristics, dosage forms, and number of chemotherapy cycles used in the different studies.

**Table 4:** Characteristics and toxicity profile of the additional studies

Author(s) (Year)	Study Design	Sample Size	GC Regimen	Toxicity				
				Neutropenia, n (%)	Thrombocytopenia, n (%)	Anemia, n (%)	Nausea/Vomiting, n (%)	Diarrhea, n (%)
van de Putte EE et al. 2016 [11]	Retrospective	51	4 cycles	2 (5.1)	0 (0.0)	1 (2.6)	1 (2.6)	–
Matsubara N et al. 2013 [58]	Retrospective	25	4 cycles	10 (40.0)	10 (40.0)	8 (32.0)	–	–
Kaneko G et al. 2011 [59]	Retrospective	22	4 cycles	6 (14.3)	9 (21.4)	1 (2.4)	0 (0.0)	0 (0.0)
Khaled HM et al. 2014 [60]	RCT	59	3 cycles	3 (5.2)	3 (5.2)	2 (3.4)	22 (40.0)	1 (1.7)
Herchenhorn D et al. 2007 [61]	Prospective	22	3 cycles	7 (33.3)	1 (4.8)	0 (0.0)	6 (28.6)	–

RCT, randomized control trial; –, not available

According to our data, patients receiving 4 courses of the GC regimen with cisplatin dose intensification had significantly higher rates of pCR and pDS compared with patients receiving 3 courses of the GC regimen. OS and CSS were similar, and toxic side effects were manageable with both treatment regimens. Therefore, we suggest using a 4-cycle NAC plan for the GC regimen. Patients who cannot tolerate chemotherapy can stop receiving NAC after three cycles of the GC regimen without affecting the treatment’s effectiveness.

Our study has several limitations. First, only three studies met the criteria, and all were retrospective. Lack of studies, particularly prospective studies, would result in low levels of evidence. Second, some of the results in our study remained significantly heterogeneous, possibly due to different protocol doses and inclusion population bias. Third, the median follow-up time is relatively short, and a longer follow-up may help to estimate long-term survival data more precisely. In future work, further studies are warranted to determine the optimal number of cycles of neoadjuvant GC chemotherapy for MIBC patients.

## Conclusions

In our meta-analysis, 4-cycle GC was superior to 3-cycle GC with regards to pCR and pDS, suggesting that 4-cycle GC is more effective than 3-cycle GC for MIBC, from pathology perspectives. The two regimens had similar survival rates, recurrence rates, and adverse effects.

However, this finding should be interpreted with caution because of the inherent limitations of retrospective studies. Large-scale RCTs and long-term follow-up studies are warranted to validate these outcomes.

## Data Availability

All data analyzed during this study is included in this published article.
